# Supplementary material for: An Interaction Network of RNA-Binding Proteins Involved in Drosophila Oogenesis
Source: Mol Cell Proteomics. 2020 Nov 25;19(9):1485–502. doi: 10.1074/mcp.RA119.001912 (PMC8143644; doi:10.1074/mcp.RA119.001912)

Raw file	Scan	Method	Score	m/z
20170411_CO_0685FuBo_R20R21R22_Mix2	6889	FTMS; HCD	77.6	439.25

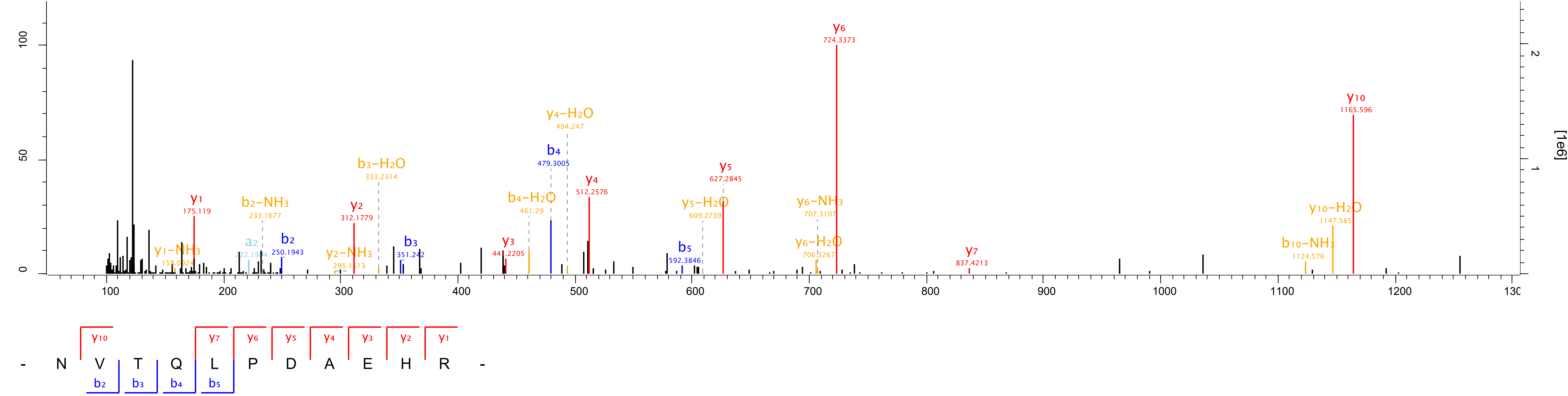

Supplement: Supplementary file 1 [file mmc1.zip › mmc1/157750_1_supp_536530_qry2q7.zip!/Single_peptide_identifications_R17-R22/20170411_CO_0685FuBo_R20R21R22_Mix2_6889.pdf]
